# “Shutdowns Are Like You’re Stuck on the Blue Screen of Death”: A Metaphor Analysis of Autistic Shutdowns

**DOI:** 10.1089/aut.2024.0193

**Published:** 2025-02-12

**Authors:** Katie Paris, Alexandru “Zeph” Lodestone, Melissa Houser, Laura Foran Lewis

**Affiliations:** 1 University of Vermont College of Nursing and Health Sciences, Burlington, Vermont, USA.; 2 All Brains Belong, VT, Montpelier, Vermont, USA.

**Keywords:** autism, Autistic adults, shutdown, figurative language, participatory research, qualitative

## Abstract

**Background:**

Autistic adults commonly experience shutdowns, which are distressing events widely discussed within Autistic communities. Yet, few studies to date have explored firsthand accounts of shutdown experiences. In this study, we aimed to identify figurative language that Autistic adults use to describe shutdowns, thereby broadening the understanding of and providing language for this complex phenomenon.

**Methods:**

We used a participatory research approach to conduct a secondary analysis of qualitative data from two prior studies on shutdowns in Autistic adults. Primary studies used asynchronous interviews and a survey, with a total sample size of 86 participants. We analyzed a corpus of 87 typed pages using the Pragglejaz Group’s metaphor identification procedure to identify figurative language that Autistic adults used to describe shutdowns.

**Results:**

Autistic adults in this sample used six metaphors to explain shutdowns, including the following: being frozen, a computer crash, going inside myself, when I can’t keep up, survival mode, and playing a role.

**Discussion:**

These metaphors help to externalize the internal experience of shutdowns. Participants described shutdowns as a state of being stuck, often involving physical and/or vocal immobilization. They frequently described shutdowns as a response to perceived threats, connecting them to a sense of survival and self-protection. Metaphors provide a rich context for understanding and talking about shutdowns. Findings also highlight how Autistic adults organically construct metaphors to articulate their experiences. Future studies could use metaphor analysis to explore other affective experiences of Autistic individuals.

**Community Brief:**

**Why is this an important issue?**

Many Autistic people experience shutdowns, but there is not much research on what shutdowns feel like. Studies that have looked at shutdowns found they can be hard to explain, especially to people who have not had a shutdown before. People often describe shutdowns as feeling frozen or stuck. We need to better understand shutdowns from the perspectives of people who experience them.

**What was the purpose of this study?**

We wanted to understand how Autistic adults describe shutdowns. Metaphors can help explain an experience by comparing it with something else. Our goal was to find out what metaphors Autistic adults use to describe shutdowns and if those metaphors can help explain what a shutdown feels like.

**What did the researchers do?**

In two earlier studies, we asked 86 Autistic adults about their experiences with shutdowns. Some answered questions in interviews, while others filled out surveys. Using these data, we used a method called “metaphor identification procedure,” meaning we looked for words or phrases where participants used metaphors or other figurative language to compare their experience with a shutdown with something else. Once we identified these metaphors, we grouped them together to find metaphors that summarized the experience for participants.

**What were the results of the study?**

We found six common metaphors that participants used to compare shutdowns with something else to help explain their experiences. Participants compared shutdowns with the following: being frozen, a computer crash, going inside myself, when I can’t keep up, survival mode, and playing a role.

**What do these findings add to what was already known?**

Few studies have looked at what it feels like to have a shutdown for Autistic people. Our findings add a rich description of what shutdowns feel like and add Autistic voices to the literature.

**What are potential weaknesses in the study?**

Most participants in this study were White, so we are not sure if these findings are relevant for people of other racial backgrounds. Since people needed internet access to take part in this study, we may also be missing the perspectives of people who do not have access to a computer or internet.

**How will these findings help Autistic adults now or in the future?**

These findings give non-Autistic people a better way of understanding what shutdowns are like for Autistic people. This knowledge can guide future research and help clinicians better support Autistic people who experience shutdowns.

## Introduction

Autistic individuals in online communities have frequently discussed shutdowns as a common and distressing experience, yet few studies have explored these events from Autistic perspectives. Shutdowns often occur as a stress response, similar to the experience of meltdowns but experienced internally, involving withdrawal, exhaustion, and emotional pain.^1–4^ These events range from mild to severe and can interfere with executive functioning, communication, motor movement, volition, and emotional well-being.^4^ Based on clinical observations, Shah^5^ identified shutdowns as “a temporary shutdown of social interaction and communication at times of acute severe stress and anxiety,”^(p25)^ noting that frequent and prolonged shutdowns, along with other catatonic signs, could escalate into full catatonia.

Researchers have increasingly focused on understanding and differentiating between Autistic shutdowns and related phenomena, such as Autistic burnout, inertia, and meltdowns, as the consequences of these experiences on the lives of Autistic people have become more evident.^4,6^ Phung et al.^4^ took a critical step in defining these constructs using a reflexive thematic analysis to analyze interviews with eight Autistic children and youth about their experiences with burnout, inertia, meltdown, and shutdown. Participants described shutdowns as “feeling frozen,” which included physical components such as feeling exhausted, slow, or physically stuck; cognitive components such as difficulty making decisions, focusing, and communicating; and emotional components such as feeling frustrated at being unable to fulfill tasks. However, Phung et al. noted that shutdowns were less emphasized in interviews with children and youth compared with meltdown. They hypothesized that shutdowns might serve as a coping strategy for adults to avoid meltdowns, as shutdowns carry fewer perceived social consequences than meltdowns, which are more outwardly expressed.

Qualitative studies exploring various other Autistic experiences, such as studies about relationships,^7^ employment,^8^ empathy,^9^ and parenting experiences,^10^ have also highlighted shutdowns. In these studies, participants described shutting down in the face of social, sensory, and emotional stressors, frequently leaving them unable to speak, move, or respond to their surroundings. This lack of response can pose risks for Autistic individuals who may be unable to remove themselves from potentially harmful situations. In addition to immediate safety concerns, shutdowns frequently occur during health care interactions, which can limit access and meaningful participation in care,^11,12^ and within the criminal justice system, where they can hinder communication and contribute to systemic inequities in legal outcomes.^13,14^

While literature on firsthand accounts of burnout^15,16^ and inertia^17,18^ is expanding, qualitative research delving into Autistic experiences of shutdowns remains scarce. In two prior studies, our team began exploring shutdown experiences among Autistic adults. In the first study, we used descriptive phenomenology to explore the lived experiences of shutdowns^19^ and meltdowns^20^ for 32 Autistic adults. We found that shutdowns were diverse in their expression but were generally triggered by social, sensory, informational, or emotional stressors. Shutdowns involved intense negative feelings, loss of control, inability to respond to surroundings, and a focus on survival and meeting basic needs. Meltdowns, triggered by similar stressors, involved extreme emotions and losing a sense of logic and control.^20^

In the second study,^21^ we developed and piloted a communication tool for Autistic adults to use with health care providers to talk about meltdowns and shutdowns. While we intended to explore the psychometric properties of this tool in addition to open-ended feedback, participant responses indicated that the tool was problematic and not acceptable in its current form. Thus, our analysis shifted to explore why the tool was not acceptable, which elucidated ableist assumptions within our tool. Due to our research aim, we did not analyze open-ended comments about shutdown experiences in our initial study.

While our findings from these prior studies provide some preliminary insight into shutdown experiences, further study is needed to understand and translate the complexity and diversity of these experiences for allistic audiences and to develop a shared language for talking about them. In an ethnographic investigation of Autistic adults’ experiences with bodily sensations and their relationship to their Autistic identities, Belek^1^ explored how members of autism-related social groups integrate autism concepts, such as shutdown, into their identities and their understanding of their bodies. Belek described the difficulty of one participant trying to find words to describe a shutdown experience, sharing, “…the inadequacy of language looms as I was effectively asking her to ‘birth a new language.’”^(p36)^ Belek compared his experience with this participant to a study about chronic pain by Good,^22^ in which Good shared that a participant “struggles to put into words an experience that resists language, a primal experience … that is at once ultimately real and ultimately indescribable.”^(p30)^

Metaphor analysis is a methodological approach to describe the indescribable. This method has been successfully applied to explain harrowing, complex, or isolating phenomena to an audience who has no lived knowledge or experience of these events, including traumatic birth experiences,^23^ anorexia,^24^ and early-onset dementia.^25^ Given that shutdowns are frequently internal and not outwardly observable, metaphors used by Autistic adults can translate these experiences into visceral, concrete language, which can help “externalize” this experience for non-Autistic audiences. Furthermore, this language can help other Autistic people make sense of their own experiences. In this secondary qualitative analysis, we aimed to identify metaphors used by Autistic adults when describing shutdowns, fostering deeper insight and understanding of this experience.

## Methods

The Neurodiversity Research Partnership, a team of Autistic and non-Autistic academic and community members, conducted this study using a community-based participatory research approach.^26^ Community partners participated in the development of the study design, interpretation of findings, and dissemination. Following the Academic Autism Spectrum Partnership in Research and Education (AASPIRE) practice guidelines,^27^ team members shared power throughout the research process. The first author, a student, did not receive compensation for this study. The second and third authors, community partners, received compensation through discretionary funds since we did not have formal funding for this study.

As we consider our positionality and its potential influence on our findings, we acknowledge our personal lived experiences with shutdowns as well as burnout, ableist discrimination, and trauma. Our involvement in academic, clinical, and community autism groups as well as our relationships with neurodivergent family members, friends, patients, and colleagues also shapes our perspectives. While we aim to separate our personal experiences from the accounts of our participants, we recognize that these experiences influence our worldviews. We approach our findings with an identity-affirming lens and with a deep trust in the lived experiences shared by our participants.

### Research design

We aimed to explore how Autistic adults use figurative language to describe their experiences of “shutdowns.” Pioneering cognitive linguists in the study of metaphor, Lakoff and Johnson^28^ defined metaphor as “understanding and experiencing one kind of thing in terms of another.”^(p5)^ They stressed that metaphors are more than words, but are also tools for influencing thought and behavior. Levitt et al.^29^ posited that metaphors better capture an emotional experience and “evoke an experiential response in the listener … than an adjective or emotional label.”^(p24)^ Kövescses^30^ asserted that metaphor “not only pervades the language people use about the emotions, but also that it is essential to the understanding of most aspects of the conceptualization of emotion and emotional experience.”^(p20)^ By using metaphors, humans can communicate difficult or emotionally complex ideas in ways that invite more universal understanding and empathy. In this study, we applied metaphor analysis to interviews and open-ended survey data collected from Autistic adults in two prior studies.

We used a constructivist paradigm to conduct this secondary data analysis to explore how participants talk about their shutdown experiences. Constructivism emphasizes the idea that participants construct their own realities through their lived experiences, which are situated in and interact with their social environment.^31^ Thus, we recognize that the metaphors that Autistic adults use not only describe their experiences but also provide information about how they make sense of shutdowns within their lives. In addition, as researchers, we acknowledge that our own life experiences affect the ways that we understand and interpret data. Constructivism allowed us to explore the ways that individual experiences varied across contexts and to construct a shared language for talking about shutdowns.

#### Primary study 1

In the first primary study,^19,20^ we used descriptive phenomenology to answer the research question: “What is the lived experience of having a meltdown or shutdown for Autistic adults?” We recruited participants from social media and online forums related to autism. Participants reviewed an information sheet and provided electronic consent. We collected data from November 2017 to August 2018 via asynchronous interviews, in which we asked participants to describe their experiences with meltdowns and shutdowns in open-ended text boxes on a secure survey platform. Once submitted, we reviewed these responses and sent individualized survey links to participants to ask follow-up clarifying and probing questions, which continued until we felt that we had a clear understanding of participants’ responses. We sent follow-up questions in any case in which we felt that data were “thin,” or not adequately robust to understand the participant’s experience (e.g., “Can you tell us more about X?” or “Please tell us about a recent shutdown that you experienced.”), in cases in which participants described a series of chronological events or a physiological description of a shutdown without also describing their feelings surrounding it or vice versa (e.g., “Can you tell us more about what you were *feeling* during the shutdown?” or “What would this shutdown look like to someone watching from the outside?”), or in cases in which the meaning of a response was not clear to us (e.g., “Can you tell us more about what you meant by X?”). We continued data collection until “saturation,” or when themes became repetitive and no new themes were emerging from new data. In all, 32 individuals participated who identified as Autistic, were 18 years or older, and had experienced a meltdown or shutdown as an adult.

#### Primary study 2

In the second primary study,^21^ we used a mixed-methods survey design to evaluate a communication tool that health professionals could use to talk to Autistic patients about meltdown and shutdown experiences, called the *Meltdown and Shutdown Scale for Autistic Adults (MSSAA)*. We aimed to answer the research question: “Is the MSSAA an effective tool to capture key information about stress responses among Autistic adults?” We recruited participants from social media and online forums related to autism. Participants were eligible to participate if they were 18 years or older and identified as Autistic. Since we did not collect any personal identifying information, we provided an information sheet to participants and their completion implied consent. Participants completed an online survey via a secure survey platform. Participants answered Likert-type questions about their level of agreement with statements about their meltdown and shutdown experiences, such as specific triggers, behaviors, or ways that they appear on the outside during these experiences, thoughts or feelings they have on the inside during these experiences, and the frequency and impact of meltdowns and shutdowns in their life. After each section of the survey, participants had the opportunity to add comments in an open-ended text box, in which participants shared details about their personal experiences with shutdowns as well as feedback on the tool itself. We collected data from June 2021 to November 2021. We stopped data collection after 54 responses, once we determined that the tool was not acceptable based on qualitative feedback about the tool.

### Secondary analysis

We utilized an analytic expansion approach to secondary data analysis, meaning that data were used to answer new questions that emerged from conducting the primary analyses.^32^ Our research question for this secondary data analysis was as follows: “What are the metaphorical expressions used by Autistic adults to describe their shutdown experiences?” The University of Vermont Institutional Review Board approved this study. We combined deidentified qualitative data from the two primary studies, including all responses related to shutdowns in Primary Study 1 and all open-ended comments related to shutdowns in Primary Study 2, into a single dataset for analysis, yielding a corpus of 87 typed pages.

**FIG. 1.**
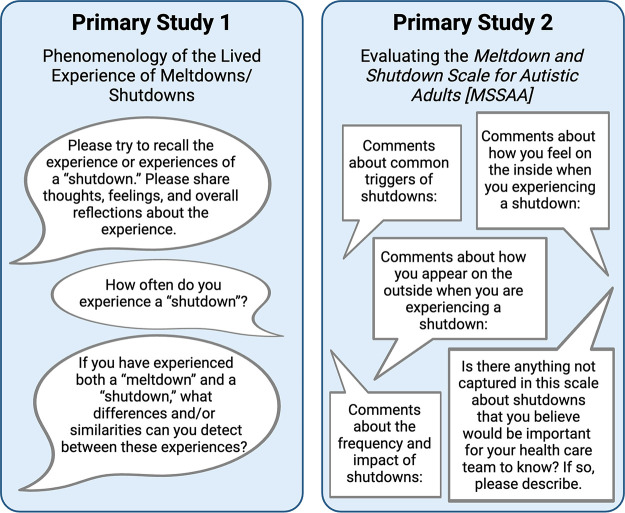
Interview and survey questions from primary studies included in secondary analysis.

From the first primary study, 26 of the 32 participants described shutdown experiences. From the second primary study, 24 of the 54 participants provided open-ended feedback related to shutdown experiences. Together, these primary studies yielded a total sample size of 50 participants for the present study. Figure 1 identifies interview and survey questions from the primary studies that we utilized for this analysis.

### Data analysis

We analyzed data using the metaphorical identification procedure (MIP), a reliable process for identifying metaphorical language developed by the Pragglejaz group.^33^ We expanded this method to include similes, which are “comparison[s] of one thing with another, usually in regard to a particular attribute, especially as a figure of speech,”^34^ as our interest was in the use of figurative language by Autistic adults more broadly. We did not code the words “like” or “as” as metaphorical.

**FIG. 2.**
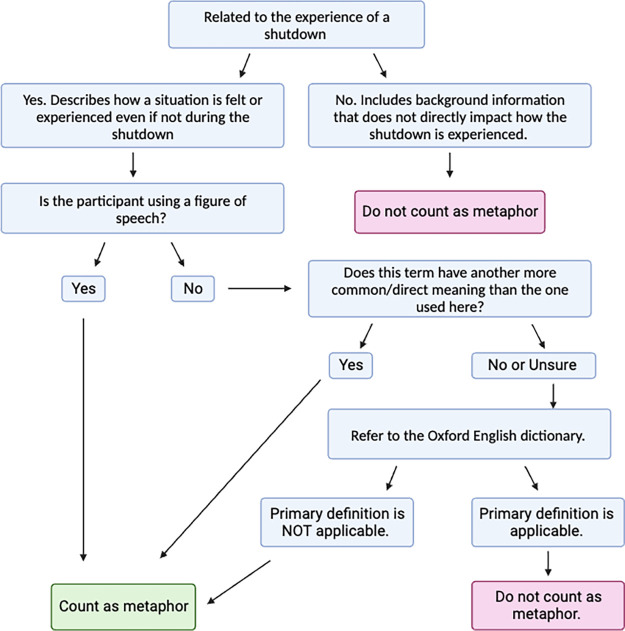
Procedure for identifying metaphor.

Following MIP, we first read all data transcripts to establish a thorough understanding of the dataset. Then, we divided the text into lexical units, or the most basic element of language that carried meaning (single word or phrase). For each lexical unit, we considered its meaning in the context of its use to determine if it was being used figuratively or not. We identified a unit as figurative language if there was a more basic contemporary meaning of the word or phrase than the one given, such as a meaning that was “more concrete [what they evoke is easier to imagine, see, hear, feel, smell, and taste; related to bodily action; more precise (as opposed to vague); [or] historically older.”^33^^(p3)^ In cases where we were unsure, we referred to the *Oxford English Dictionary* to see whether the primary definition applied to the context in which the lexical unit was used. Figure 2 illustrates the decision tree we used for MIP.

Here is an example of how we applied MIP:

I’ll/only/notice/way/later/that/I’ve/been/mostly/on/autopilot,/not/feeling,/just/“acting,”/for/hours/sometimes.

This sentence contains 18 words (or lexical units), and we identified two of them as metaphor: *autopilot* and *acting.* The first and last author independently coded lexical units, then compared and discussed. If we were not able to reach consensus, we discussed these units with all authors to make a final determination.

Using MIP, we judged 98 lexical units to be metaphors in the secondary analysis corpus. Once we identified all metaphors, the first author grouped metaphors by the most frequently occurring ideas used to describe the experience of shutdown. All authors reviewed and discussed groupings and corresponding lexical units to ensure that metaphors were grouped in ways that were representative of their derivative quotes and meanings.

### Trustworthiness

We used Lincoln and Guba’s^35^ evaluative criteria to establish trustworthiness in this study. We established credibility through triangulation of data sources with the inclusion of multiple studies in this analysis and by adopting a participatory approach. Autistic research team members provided valuable and informative feedback through every step of the research process and were crucial to framing, understanding, and interpreting responses in this dataset. To establish transferability, we included thick descriptions of participant accounts through use of extended quotations. We established dependability through a detailed audit trail. Our team created and utilized a collated list of operational definitions and procedures with examples of metaphors and lexical units to guide decision-making throughout the metaphor identification process. To establish confirmability, all researchers maintained reflexive journals to acknowledge and explore personal biases, which we also discussed as a team throughout the research process. Our team also engaged in frequent discussions about our individual interpretations of the data, which allowed us to explore multiple ways of understanding the figurative language identified.

## Results

Table 1 provides demographic information about this sample. In total, we identified six metaphors that summarize the figurative language participants used to describe the experience of having a shutdown, including *Shutdown is*: being frozen, a computer crash, going inside myself, when I can’t keep up, survival mode, and playing a role.

**Table 1.** Demographic Characteristics of Sample (*N* = 86)

	*n*	%
Age		
Mean: 35.2 years (missing 8)		
Range: 18–69 years		
Gender		
Agender	2	2.3
Female	43	50.0
Gender fluid/gender queer/non-binary	12	14.0
Male	15	17.4
Missing	14	16.3
Self-reported diagnosis		
Formal evaluation and diagnosis by a health professional	51	59.3
Informal evaluation by a health professional	12	14.0
Self- or other-diagnosed	13	15.1
Missing	10	11.6
Highest level of education		
Some high school	1	1.2
High school graduate	10	11.6
Trade/technical/vocational training	4	4.7
Some college	18	20.9
College graduate	19	22.1
Some postgraduate work	6	7.0
Postgraduate degree	18	20.9
Missing	10	11.6
Employment status		
Employed, full-time	28	32.5
Employed, part-time	11	12.8
Student	9	10.5
Homemaker	4	4.7
Retired	2	2.3
Unable to work/disabled	10	11.6
Unemployed	12	14.0
Missing	10	11.6
Race		
Asian/Pacific Islander	1	1.2
Black or African descent	3	3.5
Multiracial	1	1.2
White	67	77.9
Missing	14	16.2
Ethnicity		
Hispanic or Latino/a/x/e	5	5.8
Not Hispanic or Latino/a/x/e	69	80.2
Missing	12	14.0

### Shutdown is: Being frozen

Participants repeatedly described shutdowns as feeling “cold” or “frozen.” For some, this was a sensory experience akin to a physical temperature change. One respondent described, “…you feel freezing cold, but you can’t escape, and you don’t have the proper cold weather gear either.” Another shared, “Feeling of being in freezing cold, shivering.”

Others used the word “frozen” to describe an experience in which they felt “stuck,” describing a loss of mental autonomy that manifested in the form of inability to think or communicate needs, as well as physical autonomy, experienced as immobility. One participant stated, “I freeze up almost completely. Verbal communication becomes very difficult beyond simple acknowledgment or short answers to concrete questions. Movement takes lots of effort.” Another shared:

I get stuck in a mental loop of trying to process each scenario, but they come too quickly and cause enough anxiety, that I can’t decide which thoughts to prioritize, and I freeze while my brain is trying to sort it out. … My body freezes while my brain tries to decide what to do …

Participants shared that they “lose the ability to speak” and “I was physically unable to think.” One described, “Impossible to move. Impossible to think. Impossible to cry. I can’t get up … It’s like the brain and the body will not work.” Participants often described the mind and the body as separate entities that each experienced being “frozen” or “stuck” distinctly from each other but also mutually influencing one another. For example, the body was often described as being stuck as a secondary consequence to the brain being stuck, and the sensation of being physically stuck raised alarm and compounded feelings of being mentally overwhelmed.

This “frozen” state often contributed to feelings of helplessness and distress. The accompanying physical immobility, confusion, and psychological distress from a shutdown are illustrated in this response: “I freeze like a statue and can’t move by myself. I feel more overwhelmed than can even be described and completely bewildered. There are tears, it’s hard to breathe. I need someone to physically move me from the situation.”

### Shutdown is: A computer crash

Many participants used metaphors that compared shutdowns with computer crashes. One participant shared, “Shutdowns are like if your computer crashes and you’re stuck on the blue screen of death for hours before rebooting.” Another shared, “It’s like if you click the mouse button on your computer too many times, the computer receives many conflicting commands, and then freezes up because it can’t process the commands as fast as it’s receiving them,” and another, “I am stuck, like when a computer crashes and takes a long time to start up again.” Previous research related to technology, informatics, and theories of cognition has also identified the metaphor of the mind as a computer.^36,37^ However, participants in this study uniquely used this metaphor to describe how their brains react to stress rather than how their brains acquire, organize, or utilize information.

Participants also used other mechanical terminology to describe the shutdown experience. Many shared an inability to “function” or “process.” Others described “disconnecting” or feeling like they have “gone offline.” Participants described “turning off,” such as “a switch ‘flipped’ and I just turned off,” and “Shut down everything. Turn the world off.” One shared, “My brain feels like it shorts out and is completely empty.” Several others described a similar emptiness, using the phrase “My mind goes blank.” Again here, participants often separated the body and mind within their experience, noting that the brain stalls and thus the body becomes immobilized. Many participants described “staring off” at a blank wall or a fixed point for extended periods of time while this happened, such as “I find myself staring at blank spots, and sometimes hours will pass without my awareness of how much time has gone by.”

Like the metaphors about feeling frozen, participants expressed feelings of powerlessness in these situations. For example, one participant described: “Imagine pressing a computer’s shut[down] button on accident. And nothing you do can stop it from happening.” They frequently referred to this as a feeling of being “stuck” until they had time to “recharge.”

### Shutdown is: Going inside myself

Many participants described a feeling of leaving a situation by going to a place within themselves. Some participants shared the experience of feeling far away from others, such as, “I go completely into my World and am not in this World at all,” and “I am in a cave.” Others described a sense of “retreat[ing] inward” as a coping mechanism in response to a stressful situation, for example, “I psychologically remove myself,” and “I can sense the dynamic has changed, and I can’t process anything further, so I remove myself just so I can deal with what’s already happening in my brain.” As one participant shared, “I retreat deep into myself, like a snail way the hell inside its shell. I won’t come anywhere near the outside until I’m ready … It is indeed like going deep away from everything and everyone but my own interests.”

For some participants, experiencing shutdowns as an “internal” and “inward” event may serve as a protective mechanism, whether consciously or unconsciously, to “keep it inside” and not “freak out” or “lose it.” This response likely reflects years of living in a society that often penalizes Autistic people for outward displays of distress. One respondent described the “downward spiral” initiated by this fear:

We’ve been so badly abused, dismissed, or flat out told we’re “crazy” that often shutdowns happen because we’re scared to talk to a person/people further. That then sets into a downward spiral that may make us shutdown on ourselves … even with this understood awareness, it is hard to not shutdown on ourselves due to toxic shame from past trauma resurfacing.

Participants commonly used other words and phrases related to internalization to depict shutdowns, including “internalized meltdown,” “inner-directed,” “internally not outwardly,” “inside,” “implode,” and “implosion.”

### Shutdown is: When I can’t keep up

Participants often reported that shutdowns were triggered by feelings of no longer being able to “keep up” with surroundings. One shared:

When there is a lot to focus on at once, I have to very consciously attempt to keep up with it all at a pace that is required for that time. A shutdown usually happens when I find myself without the energy necessary to continue doing this.

Many used metaphorical language to describe the speed of incoming information, using words and phrases like “come too quickly” and “everything is moving fast,” which they often experienced before a shutdown. One shared that during a shutdown, “I am slow to respond.” Participants described this feeling of not being able to keep up as having an acute time span, typically lasting minutes to hours, before they could process information again.

Other participants described the shutdown itself as exhausting, describing an experience that continues to deplete energy and mental resources until it is over. Participants described that a shutdown is like “sitting in a stupor,” “comatose almost,” and “no energy and no motivation to do anything.” One shared, “it feels as if it takes as much energy to speak a sentence as it does to sprint a mile,” and another, “I have to keep wading through the exhaustion.” Participants recounted the need for time to be alone to “recover” and “replenish my energy levels” after a shutdown.

### Shutdown is: Being in survival mode

Participants described shutdowns as a survival instinct, as a means of self-protection in the face of danger. Many used words that portrayed a sense of defenselessness or even of terror, such as feeling “cornered,” “trapped,” and “attacked.” For example, one shared that they experience shutdowns “… if I’m cornered and cannot escape a bad situation.” Participants described bodily reactions that matched this experience, such as becoming “rigid,” “fists are clenched,” and “really stiff tight muscles.” Several described shutdowns as a response to being “forced” into undesirable or unsafe situations and echoed a need to escape. One shared, “All I can add is the feeling of ‘Escape! Get out!’ It is almost a panic attack-like reaction,” and another, “Shutdown is when I am trying to escape the world around me.” One participant described that shutdowns occur “instinctively” in situations when “…I do not feel safe.”

Participants also used the phrase “fight or flight mode” to describe a shutdown response to an unsafe situation. They described the shutdown experience as reverting to an “automatic” state where they “focus on the need for survival and meeting basic needs.” As one described, “I become hyper self-protective. … The only person I’m capable of considering is myself. … I simply lack the ability to extend myself any further and so I start disregarding those around me.”

### Shutdown is: Playing a role

Participants shared that shutdowns were often a consequence of suppressing Autistic traits (consciously or subconsciously), referred to by participants as “play[ing] a role,” “acting,” or “camouflaging.” One participant described shutdowns as a reaction to needing to hide oneself. He shared that shutdowns frequently occurred at social or family functions “because I know they don’t really want me there. Rather they want me there, as long as I don’t act, talk, or carry on like me. So … I find a place to make myself invisible.”

While most participants described playing a role as a precursor to a shutdown, some participants also described “losing access to my personality” and “acting” during a shutdown. For example, one participant described taking on a new persona as a “yes man” during a shutdown:

No agency anymore. … When I’m “shutdown”, if I’m not mute, I’m a yes man. I will agree to any plan, any offer and any request. I might not think going to a crowded restaurant is a good idea for me, but in that state, I’ll agree to it in spite of myself, potentially leading to more issues later.

Others described a similar loss of agency and emotional awareness. One described:

I lose touch with my emotional feelings. I also have less sense of being “in” my body. … It’s like the ability to notice I’m shutting down, shuts down too, and I’ll only notice way later that I’ve been mostly on autopilot, not feeling, just “acting,” for hours sometimes.

## Discussion

Figurative language is pervasive in communication and is fundamental to social interaction.^38^ Metaphor, in particular, “is an essential tool for explaining and understanding complex topics.”^39^(p23) Our findings reveal powerful metaphors that Autistic adults used to illuminate the internal experience of shutdown.

Some of the metaphors identified in our study confirm those identified by Phung et al.,^4^ including “feeling frozen” and “a slow computer” as descriptors of this experience. Participants in Phung et al.’s study^4^ also used the metaphor of a heavy blanket: “like my blanket weighs 500 pounds and it’s weighing me down.” While participants in our study did not use this language, they captured a similar experience in their descriptions of physical immobility and “wading through exhaustion.” Our findings further shed light on the painful experiences of Autistic people in their daily lives beyond specific shutdown events, including experiences of needing to “play a role” to survive, of exhaustion, and of feeling as though friends and family would prefer them to be invisible.

The “freeze” response has been identified as an adaptive response to acute stress, typically including vocal and motor inhibition.^40^ In the context of fight, flight, freeze, fawn, and flop responses, freezing is often associated with significant fear,^40^ past experiences of trauma,^41^ and anxiety.^42^ Likewise, our findings indicate that shutdowns may be a response to perceived threat, connected to a sense of survival and self-protection. Several participants in our study linked shutdowns to trauma, panic, fear, and dissociation. Our findings depict shutdowns as a cascade of mental and physical experiences that interact with and build on one another: a state of fear that leads to freezing and a sense of helplessness that comes from being frozen.

Descriptions of exhaustion in the metaphor “Shutdown is when I can’t keep up” and “Shutdown is playing a role” also mirror descriptions of Autistic burnout from previous qualitative studies, including exhaustion, withdrawal, and cognitive changes.^15,16,43^ However, participants in this study spoke to a more acute experience, often lasting a matter of minutes to hours and occasionally lasting days, whereas qualitative research on burnouts describes a chronic experience lasting months to years.^15,16,43^ Still, since the taxonomy around meltdowns, shutdowns, burnout, and inertia is still quite new, and since exposure to these terms may vary depending on participants’ engagement in Autistic communities and literature, it is possible that some participants were describing aspects of burnout rather than shutdown in the present study and lacked the language to differentiate between these distinct experiences. Continued exploration into distinguishing factors between these experiences and how best to support individuals during each of these events remains an area for needed research.

Our findings also add to the understanding of shutdown as a potential consequence of camouflaging, which is already known to contribute to burnout, anxiety, depression, exhaustion, stress, and suicidality.^44,45^ Since many Autistic people describe camouflaging as an important part of maintaining safety,^46–48^ our findings suggest that in some cases, shutdowns may be a trauma response to the danger experienced when camouflaging is no longer possible. As a largely internal response, participants deemed shutdowns to be “safer” than outward expressions of distress. Yet, while some described protective qualities of shutdowns, such as a forced focus on meeting basic needs, others identified significant safety risks posed by shutdowns, such as physical immobility, inability to communicate needs, and loss of decision-making capacity. Results highlight the potential physical and psychological harms of the shutdown experience.

### Autism and figurative language

Findings from the present study also highlight how Autistic adults organically use figurative language. Researchers often cite a difference in the understanding and use of figurative language, particularly metaphorical language, as a defining characteristic of autism. Many believe that Autistic individuals interpret and use metaphors more literally^49^ or in a different way.^50,51^

The mechanisms responsible for this phenomenon are likely complex and are not yet clearly understood.^52^ Current research provides conflicting evidence regarding whether a true difference exists, with confounding factors such as study design and differing task selection (e.g., multiple choice responses versus verbal explanation) influencing understanding of metaphorical processing and use in Autistic individuals.^53^ Multiple studies found no true differences between groups when Autistic and non-Autistic individuals were matched for verbal ability,^54–56^ while others did point to significant differences between groups in metaphor processing.^57–60^ Some evidence suggests that Autistic children and adults demonstrate a “unique verbal creativity”^51^(p6) when generating novel metaphors. In two studies, Autistic children and Autistic adults performed higher than age and language-matched peers in their ability to generate novel and creative metaphors.^50,51^

Our study adds to the current understanding of metaphor use in Autistic adults. Participants effectively used metaphors when writing about their shutdown experiences, indicating that Autistic adults can and do use metaphors in a way that can successfully communicate nuanced, emotionally complex, and difficult inner experiences. Importantly, this study used an interview method that relied only on written communication via an online survey platform, which may have played a mediating role by reducing the auditory, communication, and social demands on participants. A study by Glenwright and Agbayewa^61^ reflected these results through the use of a computer-mediated task to assess verbal irony, which is another form of frequently used figurative language. In a comparison of Autistic and non-Autistic children and adolescents, researchers found no significant differences in the accuracy of identifying and understanding verbal irony when participants used a computer interface. Researchers concluded that reducing demands on social and verbal skills can facilitate understanding of verbal irony. These results, in addition to the results of the present study, suggest that we need to examine metaphor use in the communication modality that each individual prefers or finds most natural.

### Limitations and recommendations for future research

This study had several limitations. While this sample included a diverse representation of gender and sexual identities, the majority of participants identified as White and non-Hispanic, which limits the transferability of findings to Black, Indigenous, Hispanic, Asian, and other people of color. Future studies should include active recruitment of racially and ethnically diverse Autistic adults to explore their experiences of shutdowns.

Both primary studies were disseminated and completed entirely online, which may have excluded people with physical, cognitive, or financial barriers to completing online tasks (e.g., lack of access to reliable internet and a computer). In addition, we were unable to capture nonverbal communication, such as laughter, crying, or silence, which may have deepened our understanding of how figurative language was being used in these contexts. While Primary Study 1 included back and forth conversation using follow-up questions, Primary Study 2 was conducted as a single-survey study, meaning we were not able to ask clarifying or probing questions to gain a deeper understanding of participant responses. Still, online data collection can reach a wide population of individuals who experience a phenomenon of interest and offers opportunities to engage with individuals who may prefer computer-mediated communication over face-to-face or phone communication. Future research must seek to collect qualitative data using diverse modalities to support varying communication needs.

Collecting original qualitative data is typically a vulnerable and time-consuming process for participants and often an expensive process for researchers. Secondary analysis allows researchers to maximize the value of those data. Thus, secondary analysis “provides researchers with the ability to continue to fulfill their promise to participants to use what they have shared in order to improve the care given to other individuals.”^62^(p387) We recommend that researchers explore opportunities to reuse original qualitative data to answer yet unanswered research questions.

Given the nature of secondary data analysis, we could not verify our interpretations of figurative language with the original study participants. Instead, we supported the credibility of our analysis through the lived experiences of Autistic members of our research team. The importance of including Autistic voices on the research team cannot be overstated. Autistic representation allowed our team to explore diverse perspectives, bring our biases to conscious consideration, and explore their impact on data analysis, recognize stereotypes, and produce outcomes that are relevant and applicable to a broader range of Autistic people. Future research can benefit from including Autistic people as true partners on the research team, beginning with the conception of the study and continuing through dissemination.

## Conclusion

In this metaphor analysis of secondary qualitative data, we identified metaphors that Autistic adults used to describe their experiences of shutdown to give language to a complex, deeply emotional, and difficult to describe experience. We found six metaphors, including the following: being frozen, a computer crash, going inside myself, when I can’t keep up, survival mode, and playing a role. Participants used figurative language characterized by retreat and self-preservation. Since shutdowns are largely an internalized experience, these metaphors from Autistic voices can externalize shutdowns and deepen our understanding of this phenomenon.

Furthermore, the ability to use metaphors to explain the unexplainable has potential implications for future research on affective Autistic experiences, such as Autistic burnout, inertia, meltdowns, and other complex phenomena. Metaphor analysis can provide a way to bridge the double-empathy gap by translating uniquely Autistic experiences into a language that can be readily understood by allistic people who have not shared this experience.

## Data Availability

Due to the sensitive and personal nature of qualitative research, research data from this study are not available to be shared.
